# Characterization of a wheat mutant line 1813WH presenting increased seed dormancy and longevity, and reduced pre-harvest sprouting

**DOI:** 10.1186/s12870-025-07651-y

**Published:** 2025-11-13

**Authors:** Xingyan Li, Xiaolong Li, Shasha Zhu, Bing Han, Yanping Xing, Guorong Li, Yan Yang

**Affiliations:** 1 Key Laboratory of Germplasm Innovation and Utilization of Triticeae Crops at Universities of Inner Mongolia Autonomous Region, Inner Mongolia Agricultural University, 29 Erdos Road, Hohhot, Inner Mongolia 010029 China; 2 Agriculture and Animal Husbandry Technology Promotion Center of Hailar-Hulunbuir City, the government of Dongshan, Hailar District, Hulunbuir City, Inner Mongolia 021008 China

**Keywords:** Pre-harvest sprouting, Dormancy, Longevity, Glutathione, Raffinose family oligosaccharides, TCA cycle

## Abstract

**Background:**

Pre-harvest sprouting (PHS) poses an enormous threat to wheat production; enhancing seed dormancy is an effective strategy to mitigate PHS. However, as a highly complex trait, seed dormancy is governed by polygenes and is difficult to be enhanced in genetic manipulation.

**Results:**

In this study, a wheat mutant 1813WH was generated from a PHS-sensitive line “Long 13-3778” through EMS treatment. The mutant exhibited significantly enhanced seed dormancy with a germination index (GI) of only 4.57% (wild type: 42.77%, *P* < 0.01), accompanied by altered agronomic traits including reduced plant height and tillering, prolonged reproductive period, thicker internodes, increased grain size, and higher 1000-grain weight (*P* < 0.05); seed longevity was significantly improved, maintaining stable germination dynamics under accelerated aging treatment; metabolite detection showed that its raffinose content was 3.5 times that of the wild type, glutathione (GSH) content was 9.8 times, and the GSH/GSSG ratio was significantly increased (10.74 vs. wild type 0.68); combined transcriptomic and metabolomic analyses indicated significant enrichment of glutathione metabolism, galactose metabolism (related to raffinose family oligosaccharide synthesis), and tricarboxylic acid (TCA) cycle pathways, with decreased contents of TCA cycle intermediates and differential expression of key genes in related pathways.

**Conclusion:**

This study provided evidence for the potential relationship between seed dormancy and longevity, which may have broad implications for the conservation and utilization of germplasm resources, as well as new materials for wheat breeding aiming at enhancing seed dormancy and longevity.

**Supplementary Information:**

The online version contains supplementary material available at 10.1186/s12870-025-07651-y.

## Introduction

Pre-harvest sprouting (PHS) significantly threatens global food security, leading to both yield and quality losses [1], and $1 billion of losses annually in wheat production worldwide [2, 3]. PHS in wheat is undesirable, and a great number of researches have dedicated to addressing this issue, with seed dormancy as a potential solution to alleviate PHS [1, 4]. Seed dormancy acts as the inability of a viable seed to germinate under favorable conditions and is essential for reproduction, ensuring that seeds can withstand adverse conditions until the environment is conducive for germination and plant growth [5]. Seed dormancy is a quantitative trait and is influenced by numerous endogenous and environmental factors.

The complexity of seed dormancy involves the crosstalk between dormancy and other traits, including seed longevity, which determines the viability and germination capacity of seeds over time. In the biosynthesis of abscisic acid (ABA), many genes are associated with onset and maintenance of seed dormancy [5–7]. Evidence indicates that ABA regulatory pathways impact both seed dormancy and longevity [7, 8]. There are a lot of substances that are significantly associated with both seed dormancy and longevity. Glutathione (GSH) is one of the substances involved. During seed aging and desiccation, oxidative events are typically active to preserve the seed germination capacity. Studies have shown that the production of reactive oxygen species (ROS) is highly correlated with seed imbibition [9, 10] and cell wall loosening during germination [11, 12]. The interaction between ROS and phytohormones such as ABA, gibberellin (GA), and ethylene is crucial for seed dormancy or germination [13–15]. However, excessive accumulation of ROS can lead to oxidative stress, impairing seed vigor and ultimately resulting in a loss of germination capacity [15, 16]. GSH, a widely studied antioxidant, plays a key role in maintaining the delicate redox balance in plant cells. The accumulation of GSH to remove excess ROS is significant for establishing seed longevity [17, 18], as well as promoting germination under stress conditions, as evidenced by studies indicating that an elevated level of GSH can significantly enhance the germination rates of plants exposed to drought or osmotic stress conditions [19, 20].

Another substance influencing seed dormancy and longevity is the raffinose family oligosaccharides (RFOs), including raffinose, stachyose, and verbascose. These compounds are known to play a crucial role in the desiccation tolerance and longevity of seeds by preventing crystallization, as well as in biotic and abiotic stress tolerance by acting as signaling molecules [21–23]. Additionally, some evidences suggest that RFOs are also related to the process of seed germination [21]. It is believed that the presence of RFOs can protect the viability of seeds by modulating the level of ROS [16, 22]. The breakdown of RFOs into galactose and sucrose is crucial for rapid germination of Arabidopsis, pea, and soybean [24–26].

These findings suggest a significant correlation between seed dormancy and longevity. Studies illustrating this correlation might provide insights into the mechanisms involved. This, in turn, could have significant implications for agriculture. The use of mutants is a common method in plant science, offering the advantage of a consistent genetic background, and could facilitate the isolation of specific genes involved in seed dormancy and longevity. In the present study, a dormant mutant from the widely cultivated wheat cultivar “Long 13–3778” that is susceptible to PHS was produced through Ethyl methanesulfonate (EMS) mutation, designated “1813WH”. This mutant exhibited a strong dormant phenotype along with multiple other obvious phenotypic variations, and a joint analysis of transcriptome and metabolome indicated that the mutant enhanced seed longevity. The identification of the agronomic traits of 1813WH, as well as the basic genetic and biochemical features of this mutant, is crucial for elucidating its dormant characteristics and laying a groundwork for further research, ultimately aiming to develop wheat varieties with enhanced dormancy to reduce PHS, and advancing the understanding of the correlation between seed dormancy and longevity.

## Results

### Significant phenotypic alterations in 1813WH

A dormant mutant of “Long 13–3778” was identified from the M2 pool based on germination index (GI), designated “1813WH”. The purified M_3_ line from self-pollination was obtained, and used for this study.

**Fig. 1:**
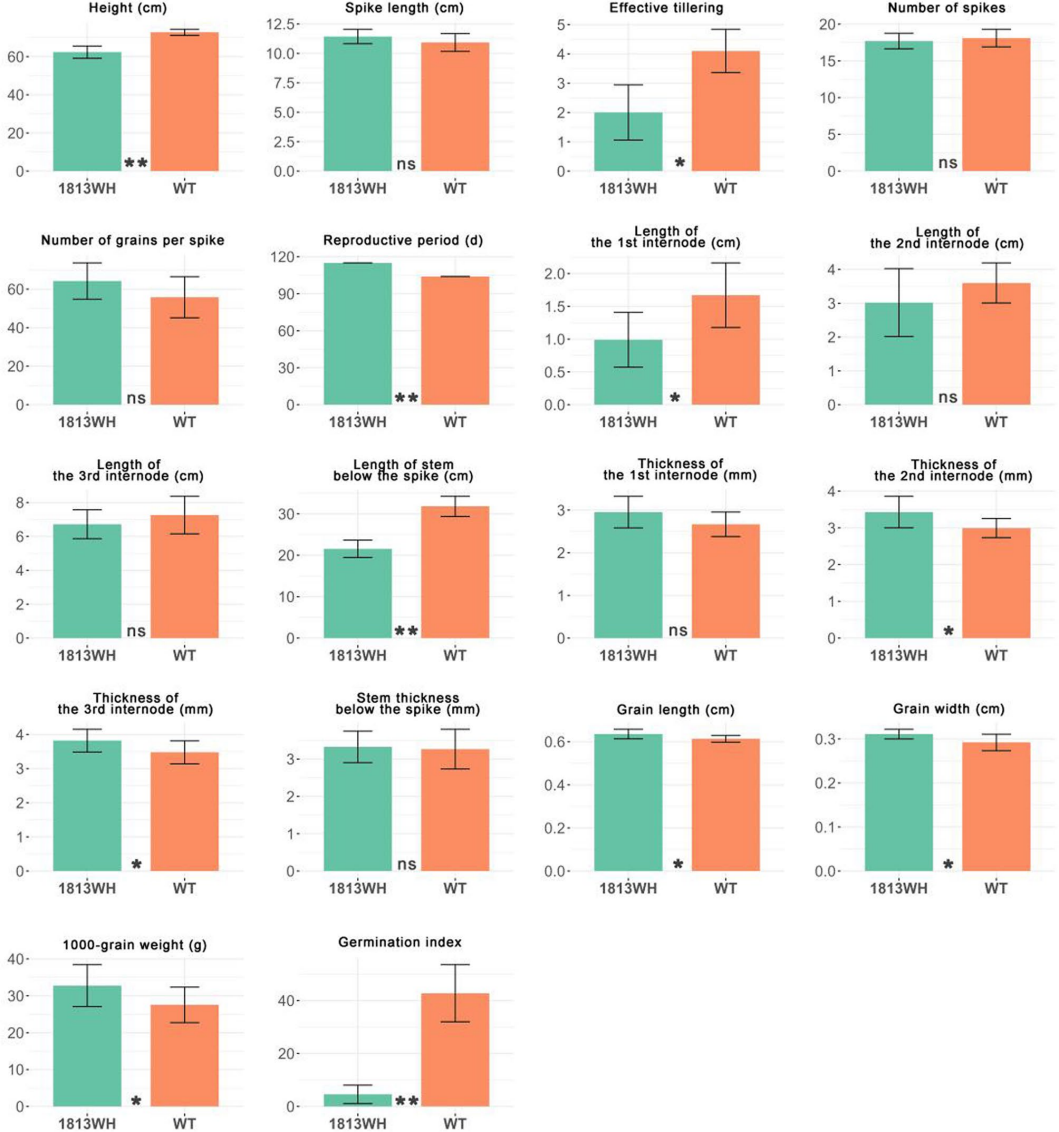
Phenotypic comparison between the mutant line 1813WH and the wild type (WT). The results of the ANOVA test are indicated within the figures. *: *P* < 0.05 **: *P* < 0.01. ns: not significant

Phenotyping the mutant and the wild type showed obvious differences in agronomic traits between two lines (Fig. 1, Supplementary Table 1). The average GI values of the wild type and 1813WH mutant exhibited significant differences, with 42.77 ± 10.79% and 4.57 ± 3.48%, respectively (*P* < 0.01). This indicates that the mutant has strong PHS resistance. The mutant displayed smaller plant size than the wild type, with significantly reduced tillering/effective tillers and shorter plant height and internodes. The average effective tillers of the mutant and the wild type were 2.0 ± 0.94 and 4.1 ± 0.74, respectively (*P* < 0.05). The average plant height of the mutant and the wild type was 62.34 ± 3.20 cm and 72.74 ± 1.59 cm, respectively (*P* < 0.05). However, the mutant showed thicker internodes than the wild type. The average second and third internodes of the mutant measured 3.43 ± 0.43 mm and 3.82 ± 0.33 mm, respectively, compared to 2.99 ± 0.26 mm and 3.48 ± 0.34 mm in the wild type. The mutant also exhibited a significantly larger grain size (Fig. 1, Supplementary Fig. 1), with the average grain length, grain-width, and 1000-grain weight being 0.64 ± 0.02 cm, 0.31 ± 0.01 cm, and 32.80 ± 5.68 g, respectively, which are significantly (*P* < 0.05) different from the corresponding measurements of 0.61 ± 0.02 cm, 0.29 ± 0.02 cm, and 27.5 ± 4.81 g, respectively, in the wild type. The mutant showed darker seed coat (Supplementary Fig. 1), and had a considerably longer reproductive period (115 d) than the wild type (104 d).

### Controlled deterioration tests reviewed enhanced seed longevity in 1813WH

**Fig. 2:**
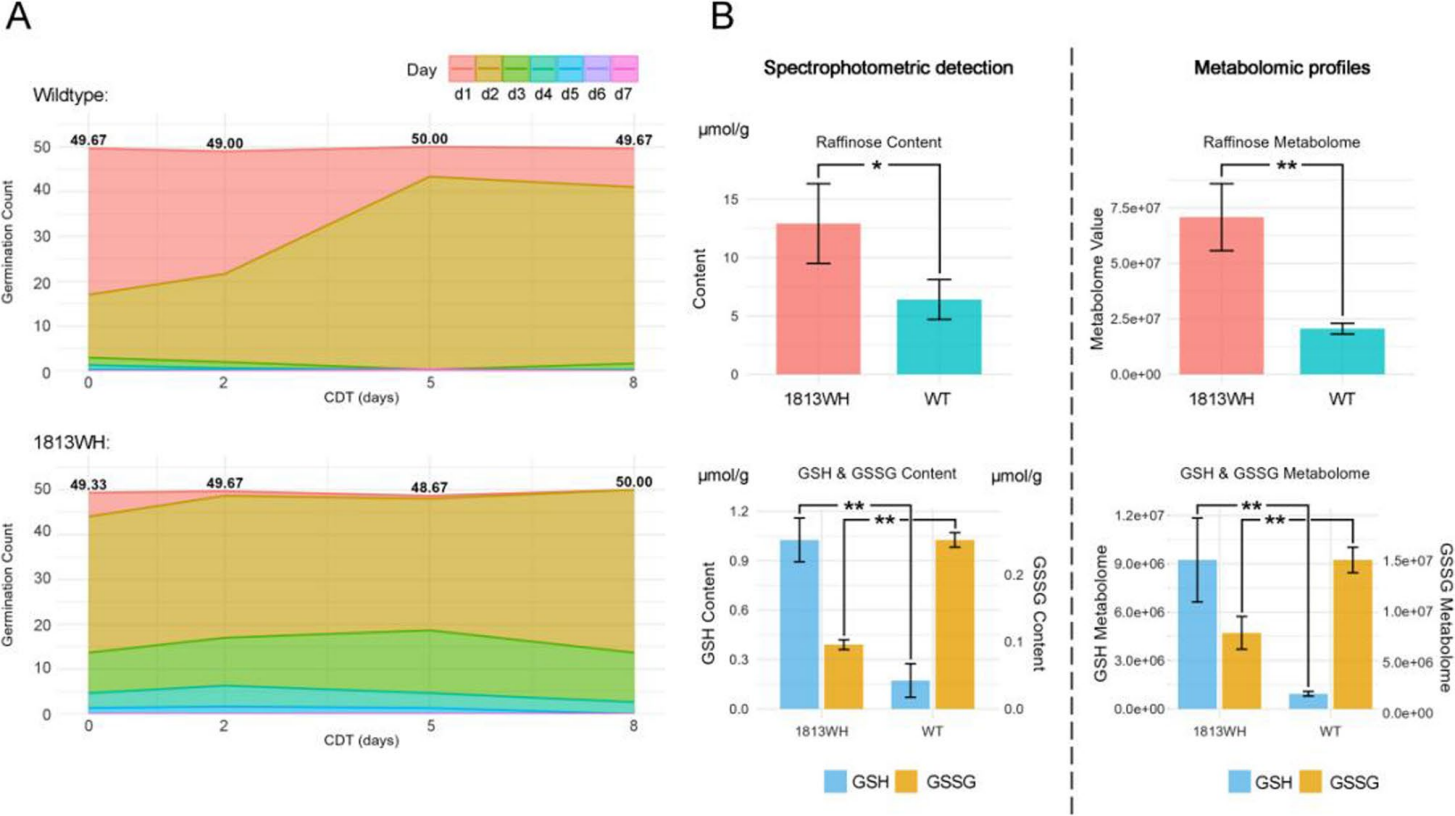
Seed quality analysis of wildtype and 1813WH strains: deterioration resistance and metabolite profiling. Average germination counts of wildtype and 1813WH strains after controlled deterioration test (CDT) treatment (**A**): the average germination counts of wildtype (up) and 1813WH (down) after zero-, two-, five-, and eight-day CDT treatment are shown; the Y-axis indicates germination counts from day one to seven under the corresponding CDT days, with the numbers above the graph representing the total average germination counts. Contents of raffinose, glutathione (GSH), glutathione disulfide (GSSG), and associated metabolomic profiles in wildtype (WT) and 1813WH strains (**B**): * and ** indicate significance levels of *P* < 0.05 and *P* < 0.01, respectively, by ANOVA test

Controlled deterioration tests (CDT) were conducted to accelerate the natural deterioration of seeds and assess their longevity (Fig. 2A). With the extension of CDT treatment duration, the germination of wild type seeds showed a delayed trend. On day one of germination, the average number of germinated seeds decreased from 32.67 ± 16.20 in the zero-day treatment group to 6.67 ± 1.15 in the five-day treatment group (*P* < 0.05 by ANOVA), and then stabilized (eight-day treatment: 8.67 ± 1.15 seeds on average). In contrast, the number of germinated seeds on day 2 increased significantly: the average germination counts in the zero-, five-, and eight-day treatment groups were 14.00 ± 10.44, 43.00 ± 1.00, and 39.33 ± 2.52 seeds, respectively (*P* < 0.01 by ANOVA). The total germination rates of wild type seeds across zero-, two-, five-, and eight-day treatments were 99.33%, 100.00%, 100.00%, and 99.33%, respectively, with no significant difference among treatments (chi-square test, *P* > 0.05), indicating that CDT treatment did not affect the overall germination rate.

In contrast, 1813WH seeds exhibited relatively stable germination patterns. On day one, the number of germinated seeds showed a potential decrease after two days of treatment but remained low overall: the average counts in the zero-day and two-day treatment groups were 5.33 ± 3.21 and 1.00 ± 1.00 seeds, respectively (*P* > 0.05 by ANOVA), and then stabilized in subsequent treatments. 1813WH seeds germination on day two consistently showed the highest germination counts across all treatment groups, with no significant variation in germination numbers with increasing treatment duration (average counts in zero-, two-, five-, and eight-day treatments: 30.33 ± 1.53, 31.67 ± 1.15, 29.33 ± 3.06, and 36.33 ± 1.53 seeds, respectively). The total germination rates of 1813WH seeds were 98.67%, 99.33%, 97.33%, and 100% for zero-, two-, five-, and eight-day treatments, respectively, with no significant effect of treatment duration on overall germination rate. The total germination rate of 1813WH was slightly lower than that of the wild type (−0.83%, *P* < 0.05 by chi-square test).

These results collectively indicate that the wild type seeds exhibited delayed germination under prolonged CDT treatment, whereas 1813WH seeds maintained stable germination dynamics, suggesting better seed longevity in 1813WH compared to the wild type.

### Raffinose, gultathione, and glutathione disulfide content indicated enhanced seed longevity and anti-oxidant capacity in 1813WH

The content of raffinose, GST and GSSG in wildtype and 1813WH seeds were determined using spectrophotometric methods (Fig. 2B). The results showed that the average raffinose content in the wildtype was 6.41 ± 1.71 µmol/g, significantly lower than the 12.91 ± 3.42 µmol/g in 1813WH (*P* < 0.05), which may confer stronger desiccation tolerance and seed longevity in 1813WH. The average GSH and GSSG contents in the wildtype were 0.17 ± 0.10 µmol/g and 0.25 ± 0.01 µmol/g (GSH/GSSG = 0.68), respectively, while those in 1813WH were 1.03 ± 0.13 µmol/g and 0.09 ± 0.01 µmol/g (GSH/GSSG = 10.74), respectively. Compared to the wildtype, 1813WH exhibited significantly higher GSH and lower GSSG levels (*P* < 0.01). This indicates stronger antioxidant capacity and potentially lower oxidative metabolism levels in 1813WH.

### Enrichment analysis of the transcriptome and metabolomics

**Fig. 3:**
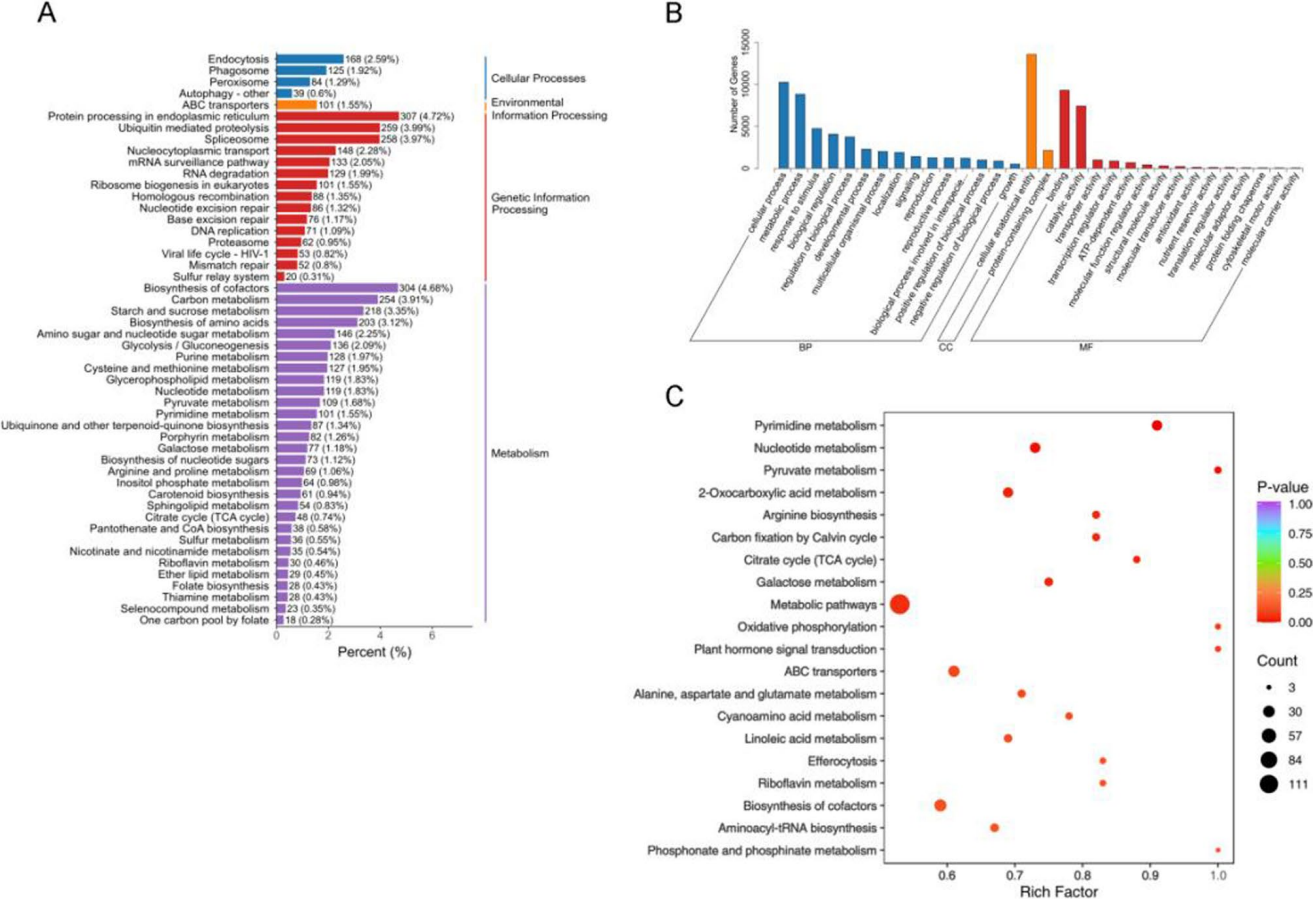
Integrated functional and pathway enrichment analysis of differentially expressed genes and metabolites in wildtype and 1813WH strains. KEGG enrichment analysis of differentially expressed genes (DEGs) (**A**), GO classification of DEGs (**B**), and KEGG enrichment analysis of differentially expressed metabolites (**C**) between the wildtype and 1813WH strains

KEGG analysis of the transcriptome revealed that genes for starch and sucrose metabolism, glycolysis/gluconeogenesis, citrate cycle (TCA cycle), and galactose metabolism were significantly enriched (Fig. 3A), indicating notable differences in energy and glucose metabolism between the mutant and the wild type. The enrichment of galactose metabolism could lead to the enrichment of raffinose family oligosaccharide metabolism, as galactose is the precursor for raffinose synthesis. In addition, the genes for cysteine and methionine metabolism were significantly enriched. This may imply alterations in sulfur metabolism, which was also significantly enriched, as cysteine is a key sulfur-containing amino acid. This result also suggests differences in glutathione metabolism between the two lines, given that glutathione is synthesized from cysteine, glutamic acid, and glycine. In addition, the genes associated with altered antioxidant activities in the mutant were also significantly enriched in the GO analysis of the transcriptome (Fig. 3B).

KEGG analysis of the metabolomic data essentially revealed similar trends in metabolic pathways (Fig. 3C). Notably, pathways related to 2-Oxocarboxylic acid metabolism, the citrate cycle (TCA cycle), galactose metabolism, and oxidative phosphorylation were found to be enriched, in agreement with the transcriptome analysis, indicating significant differences in energy and glucose metabolism between the two lines. Additionally, plant hormone signal transduction pathways were also enriched.

### Multi-omic analysis revealed enrichment in the glutathione, galactose, and TCA cycle metabolisms

**Fig. 4:**
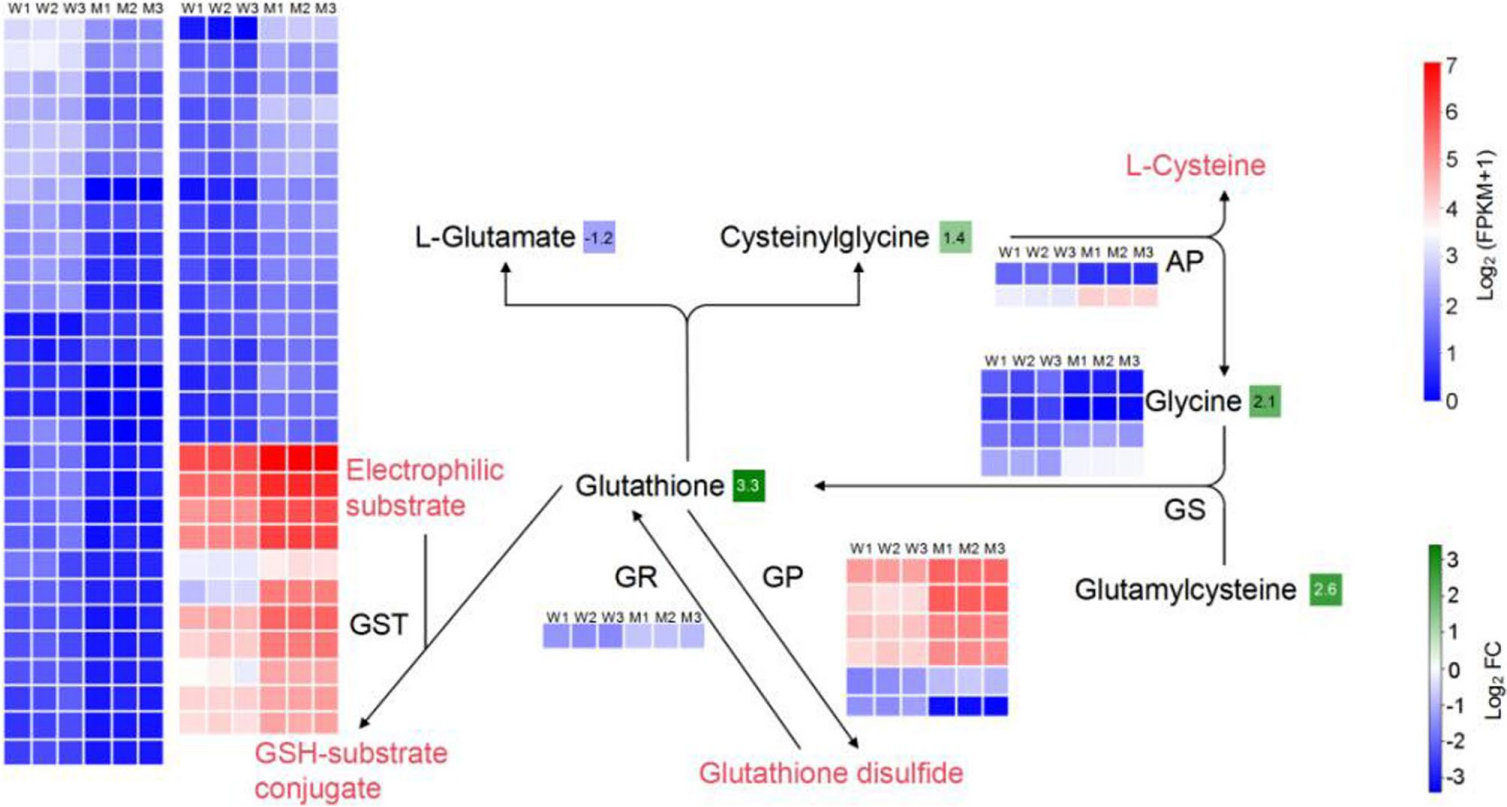
Comprehensive analysis of the genes and metabolites of the glutathione metabolism pathways. The heatmaps represent the differentially expressed genes. W1-W3 and M1-M3 represent the gene expression in the wildtype and the mutant, respectively, of the enzymes in the corresponding metabolism pathways. The block icons marking the metabolites represent the Log2 FC value of the corresponding metabolite of 1813WH compared to the wild type. The gray block represents no significant differences. The legends represent gene expression (up) and the Log2 FC value of the metabolites (down). The abbreviations represent the enzymes of the corresponding pathways. AP: aminopeptidase; GS: glutathione synthase; GP: glutathione peroxidase; GR: glutathione reductase; GST: glutathione S-transferase. The detailed differentially expressed genes and metabolomic data are listed in the Supplementary table 3, 4

**Fig. 5:**
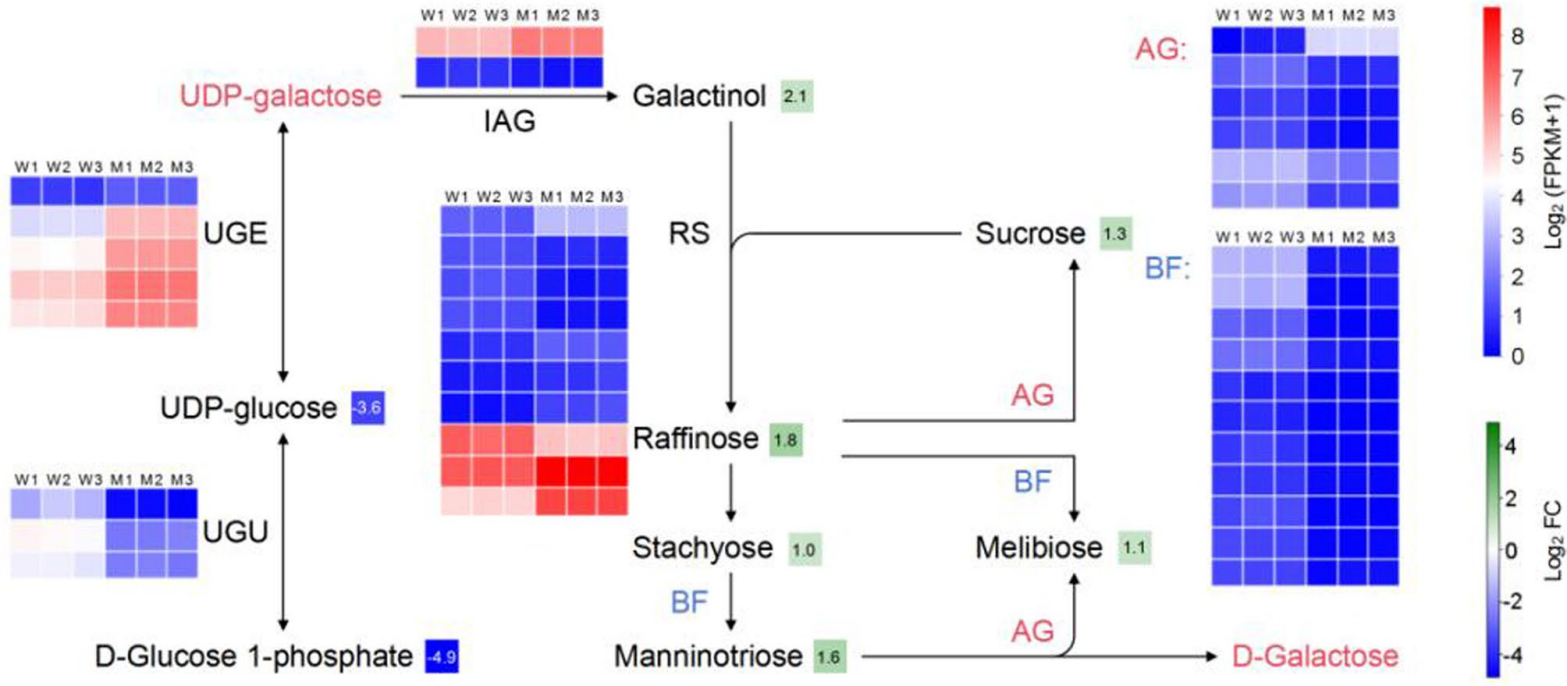
Comprehensive analysis of the genes and metabolites of the galactose metabolism pathways. The captions are identical to those in Fig. 8. The substances indicated in red text represent those that were not detected. RS: raffinose synthase; BF: beta-fructofuranosidase; AG: alpha-galactosidase; IAG: inositol 3-alpha-galactosyltransferase; UGE: UDP-glucose 4-epimerase; UGU: UTP-glucose-1-phosphate uridylyltransferase. The detailed differentially expressed genes and metabolomic data are listed in the Supplementary table 3, 4

**Fig. 6:**
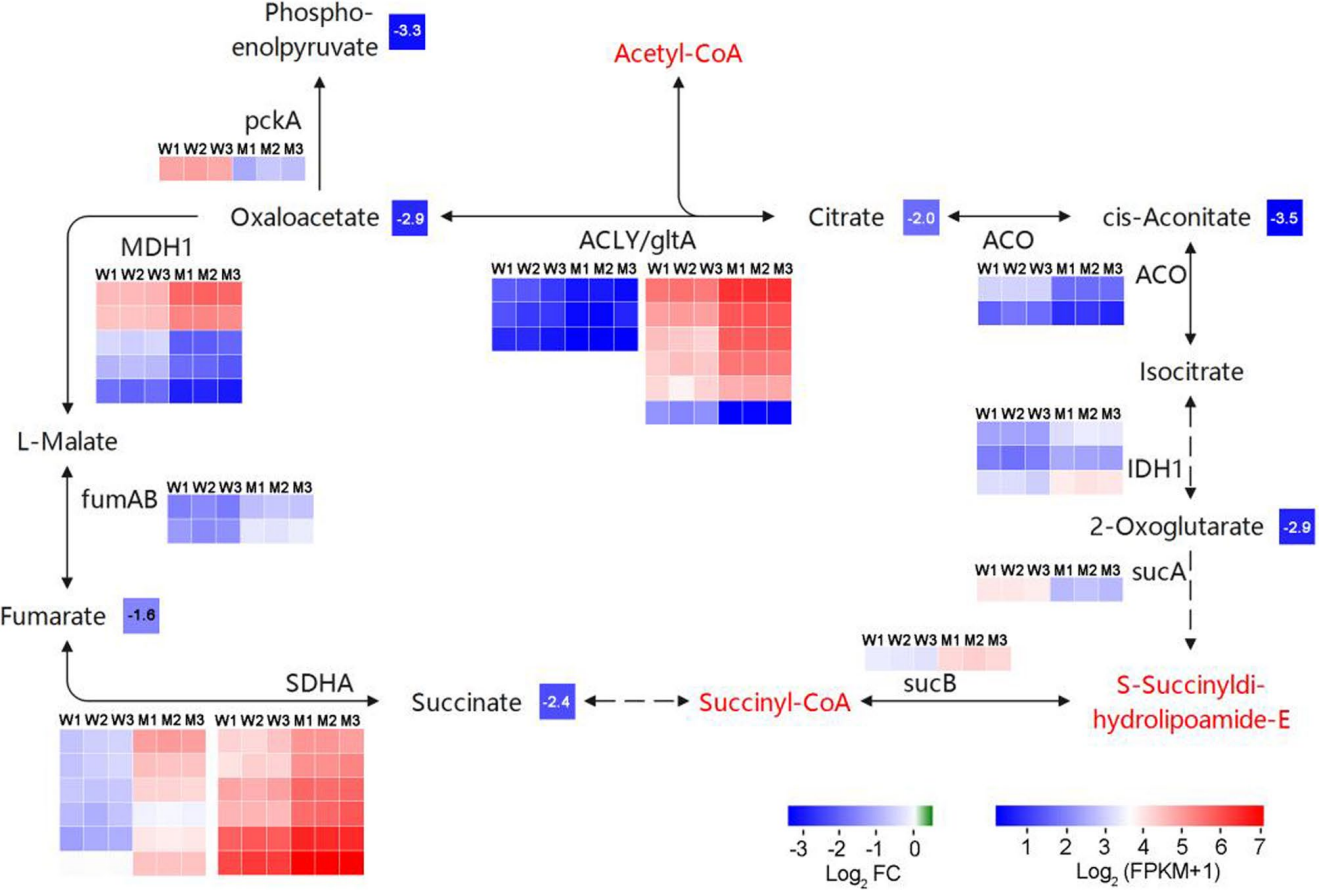
Comprehensive analysis of the genes and metabolites of the TCA cycle. The captions are identical to those in Fig. 8. The substances indicated in red text represent those that were not detected. MDH1: malate dehydrogenase; fumAB: fumarate hydratase, class I; SDHA: succinate dehydrogenase (ubiquinone) flavoprotein subunit; sucB: 2-oxoglutarate dehydrogenase E2 component (dihydrolipoamide succinyltransferase); sucA: 2-oxoglutarate dehydrogenase E1 component; IDH1: isocitrate dehydrogenase; ACO: aconitate hydratase; ACLY: ATP citrate (pro-S)-lyase; gltA: citrate synthase; pckA: phosphoenolpyruvate carboxykinase (ATP). The detailed differentially expressed genes and metabolomic data are listed in the Supplementary table 3, 4

The joint analysis of the transcriptome and metabolomics revealed intriguing insights into the glutathione (Fig. 4), galactose (Fig. 5), and TCA cycle (Fig. 6) metabolism pathways. Metabolomics revealed significant enrichment of the glutathione metabolism pathway. The content of glutathione, glycine, and glutamylcysteine was significantly up-regulated in the mutant seeds, whereas the content of L-glutamate was significantly lower than that in the wild type. The GSH content of the mutant seeds was tenfold of the wild type, which suggested a remarkable enhancement in antioxidant capacity. Moreover, genes involved in the biochemical reactions were also active. The expression levels of 57 out of 377 glutathione S-transferase (GST) genes, which catalyze the conjugation of the sulfhydryl group of GSH with numerous endogenous and exogenous electrophilic compounds, exhibited significant differences between the two lines, with all 11 differentially and highly expressed GST genes being up-regulated in the mutant. Additionally, five out of six differentially expressed glutathione peroxidase genes were also up-regulated in the mutant, suggesting enhanced conversion of GSH into oxidized glutathione (GSSG), and promoted decomposition of hydrogen peroxide. Joint analysis of transcriptome and metabolomics indicated up-regulated glutathione synthesis and corresponding biochemical pathways in the mutant seeds.

The results indicated an increased synthesis of RFOs in the mutant seeds, as metabolomics revealed an up-regulation of galactinol, sucrose, melibiose, raffinose, stachyose, and manninotriose. This was consistent with the transcriptome analysis, showing that all 11 genes with differential expression of beta-fructofuranosidase and five of the six differentially expressed alpha-galactosidase genes that catalyze the hydrolysis reaction of RFOs, were down-regulated in the mutant. Metabolomics also showed significantly down-regulated UDP-glucose and D-Glucose 1-phosphate content in the mutant, accounting for 8.2% and 3.3% of those in the wild type, respectively. Transcriptome analysis showed consistent up-regulation of five differentially expressed UDP-glucose 4-epimerase genes and down-regulation of three differentially expressed UTP-glucose-1-phosphate uridylyltransferase genes, indicating altered regulation of glucose metabolism in the mutant seeds. The accumulation of galactinol and raffinose family oligosaccharides (RFOs) in the mutant, coupled with the downregulation of genes involved in their hydrolysis, suggests a block or reduction in the breakdown of these compounds. The upregulation of UDP-glucose 4-epimerase genes may indicate a compensatory mechanism to redirect glucose metabolism towards alternative pathways, while the downregulation of UTP-glucose-1-phosphate uridylyltransferase genes suggests decreased synthesis of UDP-glucose from glucose-1-phosphate. Overall, these findings provide insights into the complex metabolic changes occurring in the mutant line, particularly in relation to RFO synthesis and glucose metabolism.

Transcriptome analysis of the TCA cycle metabolic pathway revealed that the expression levels of differentially expressed genes with overall high expression in the Citrate synthase family were significantly higher in 1813WH than in the wild type. Additionally, all differentially expressed genes in the succinate dehydrogenase (ubiquinone) flavoprotein subunit family showed significantly higher expression levels in 1813WH compared to the wild type. In contrast, the expression of genes in other families showed no consistent pattern between the two genotypes. However, metabolome analysis further demonstrated that the contents of oxaloacetate, phosphoenolpyruvate, 2-oxoglutarate, fumarate, succinate, citrate, and cis-aconitate in 1813WH were all significantly lower than those in the wild type, indicating that the TCA pathway in 1813WH may be subject to substrate limitation.

### qPCR validation

**Fig. 7:**
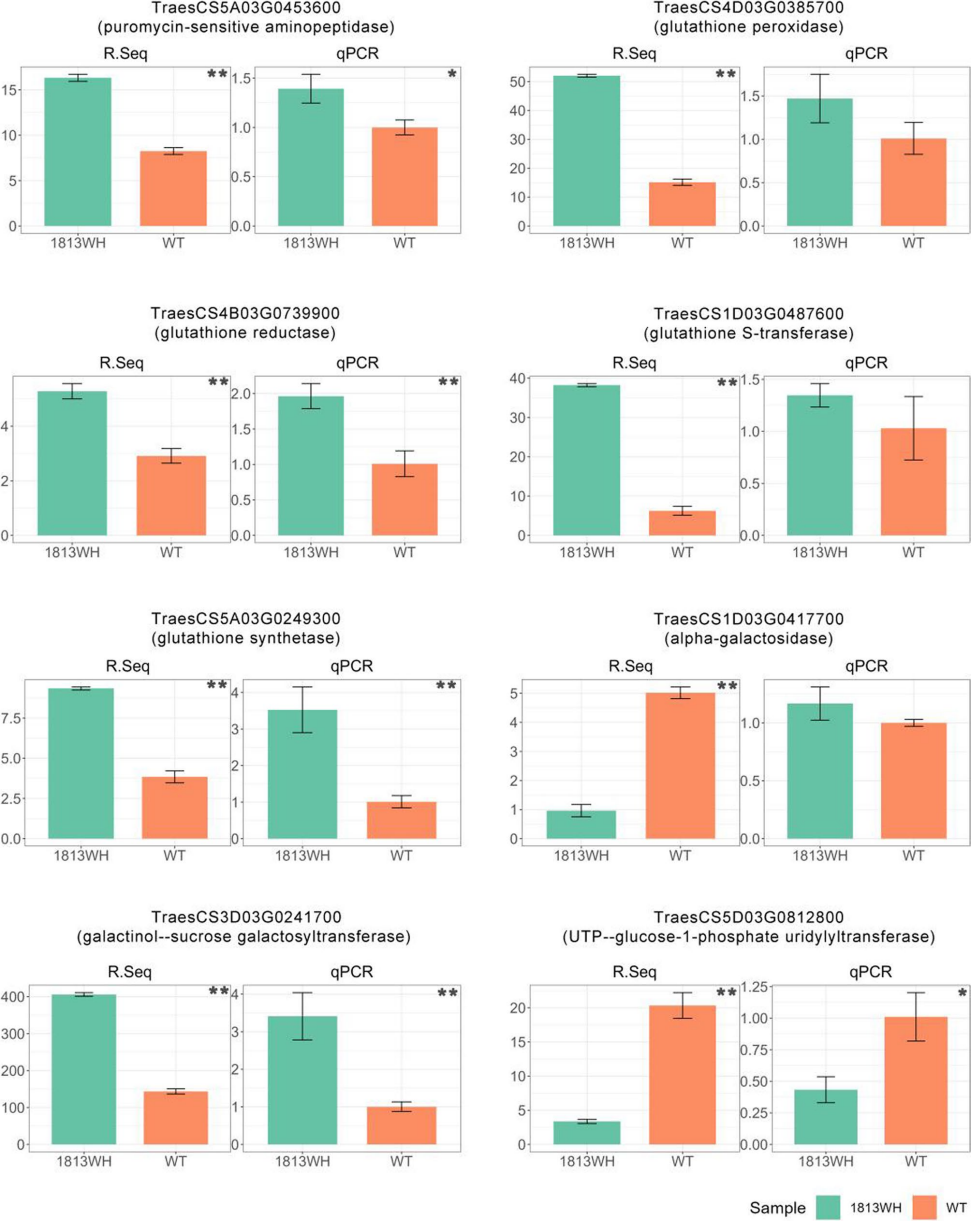
qPCR validation of RNA-Seq results for eight key genes. RNA-sequencing results and qPCR validation of eight key genes involved in glutathione or galactose metabolism pathways. The results of the ANOVA test are indicated within the figures. *: *P* < 0.05 **: *P* < 0.01. ns: not significant

Based on the above multi-omic analysis, five key enzyme-coding genes (TraesCS5A03G0453600, TraesCS4D03G0385700, TraesCS4B03G0739900, TraesCS1D03G0487600 and TraesCS5A03G0249300) involved in the glutathione metabolic pathway and three other key genes (TraesCS1D03G0417700, TraesCS3D03G0241700 and TraesCS5D03G0812800) involved in RFO metabolism, were selected for qPCR validation. These genes encode minopeptidase, glutathione peroxidase, glutathione reductase, GST, glutathione synthase, alpha-galactosidase, raffinose synthase, and UTP-glucose-1-phosphate uridylyltransferase, respectively (Fig. 7, Supplementary Table 5). The validation data showed that seven genes displayed the same expression trends in wild type and 1813WH as those observed in RNA-sequencing data except for alpha-galactosidase gene (TraesCS1D03G0417700). Notably, five of these seven genes showed statistically significant differences between the wild type and 1813WH (*P* < 0.05). These results validate the reliability of the RNA-sequencing findings, further reinforcing the confidence in subsequent research on the functions of these key genes.

## Discussion

The results in the present study indicated an enhanced antioxidant capacity due to increased levels of GSH and its associated metabolic pathways, along with reduced RFO catalysis and elevated content, suggesting increased seed longevity of the 1813WH mutant. In the meantime, the GI indicated that l813WH has a higher degree of dormancy than the wild type line. The two crucial seed traits, longevity and dormancy, for plant reproduction were closely related. Many studies indicated a positive or negative correlation between the longevity and dormancy of seeds, reflected by studies into the seed coat [27, 28], ROS production/detoxification [29–31], and plant hormones [31]. There could be a potential correlation between the two traits, as enhanced dormancy might help seeds withstand adverse environmental conditions longer, thereby promoting longevity.

The positive correlation between seed dormancy and longevity, particularly due to ROS detoxification, was not unique in our study. The loss of function of NADPH oxidase in *Arabidopsis*, leading to decreased ROS production, also exhibited a positive correlation between these two traits [29, 30]. It can be hypothesized that a moderate level of ROS is necessary to maintain the germination vitality of seeds, and excessive ROS removal could hinder germination, thus prolong dormancy. This, in turn, could enhance seed longevity by reducing the risk of oxidative damage. The relationship between RFOs and seed longevity has been thoroughly illustrated in many studies, whereas the correlation between RFOs and seed dormancy remains largely unclear. In this study, the 1813WH mutant showed an up-regulated RFO content compared to the wild type, as well as enhanced seed dormancy. This could be attributed to the anti-oxidative capacity of RFOs, as discussed earlier.

Metabolome results suggest that the TCA cycle may be limited by substrate availability, thereby impairing the capacity to generate reducing equivalents through metabolite oxidation to drive ATP synthesis, and consequently reducing energy metabolism in 1813WH [32, 33]. Since seed germination requires energy supply, reduced energy metabolism is likely to inhibit seed germination, promote seed dormancy [34, 35], and potentially lead to slow seedling growth [35], which may explain the significantly prolonged growth period of 1813WH compared to the wild type.

On the other hand, studies have shown that a decrease in TCA cycle activity triggers elevated ROS levels. This occurs because inhibition of the TCA cycle leads to electron transport chain dysfunction, resulting in increased electron leakage that directly fuels elevated ROS production [36, 37]. However, relevant studies in plants remain scarce, and given the unique metabolic constraints of seeds, further investigation is required to validate the link between the TCA cycle and ROS in seeds.

**Fig. 8:**
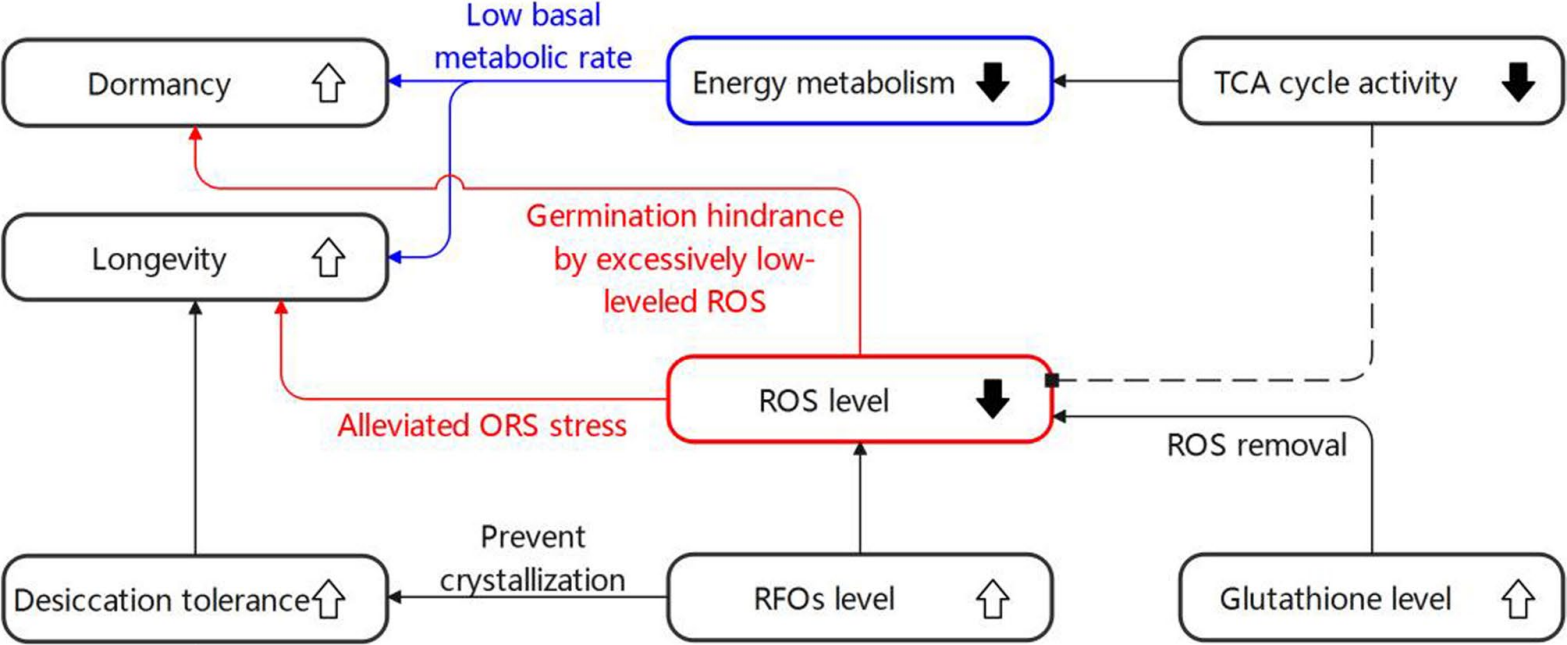
Comprehensive mechanism for dormancy and longevity enhancement in 1813WH seeds. Arrows represent enhancement (upward arrow) and weakening (downward arrow), while dashed lines indicate uncertain relationships

A comprehensive metabolic network is proposed based on the findings of the present study. The reduced TCA cycle activity in 1813WH reflects a decrease in the overall metabolic rate of the seeds, promoting their dormancy and longevity. The significantly upregulated glutathione content in 1813WH may have lowered ROS levels, alleviating oxidative stress—which could enhance longevity—and potentially hindering germination. Additionally, the significantly accumulated RFOs in 1813WH indicate enhanced desiccation tolerance and improved ROS scavenging, which would further strengthen seed dormancy and longevity. A comprehensive model of dormancy and longevity enhancement in 1813WH was proposed in Fig. 8.

In this study, the 1813WH mutant showed darker seed coat than the wild type. This may be correlated to its enhanced dormancy, as the red-grained wheat varieties possess higher dormancy than white-grained lines. In *Arabidopsis*, a mutant exhibited defects in endothelium pigmentation and increased permeability, which undermined seed dormancy [27]. Concluding from these observations, it appears that darker seed color may be positively associated with enhanced dormancy. This result has already been proven in many other studies that red wheat has stronger dormancy than white wheat [1, 38].

The 1813WH mutant exhibited enhanced seed dormancy and longevity, advantageous for crop production. However, the genetic factor responsible for these changes also led to alterations in other characteristics, such as reduced tillering, decreased plant height, thicker stems, larger grain size, darker grain color, and an extended reproductive period, some of which suggests enhanced lodging resistance (decreased height, thicker stems), while some other traits are undesirable. This raised questions among researchers about the balance between beneficial and detrimental traits introduced by genetic mutations, as well as raising the necessity of comprehensive assessments of phenotypes when evaluating the influence of certain genetic alterations, instead of focusing only on the phenotypes of interest, to ensure a holistic understanding of the impact of genetic mutation. By considering a wide range of phenotypic traits, researchers can identify potential trade-offs and develop strategies to mitigate undesirable effects.

The 1813WH dormant mutant also showed multiple phenotypic changes, including reduced plant height, decreased tillering, larger grain size, and extended reproductive period. These phenotypes have been observed to change simultaneously in numerous other studies. Rice variants carrying the *Hd1* gene exhibited promoted heading, shorter plant height, and reduced tillers [39]; wheat *TaSPL14* and *TaSPL17* mutants showed reduced plant height, shorter spikes, and smaller grains [40]; and the up-regulation of *Rht-B1b* in wheat resulted in reduced plant height and grain weight, yet increased tillers [41]. These mutations and their associated phenotypic effects highlight the complexity and diversity of genetic mechanisms that regulate plant growth and development. Understanding the genetic basis of such phenotypic changes can provide valuable insights into crop improvement strategies, enabling breeders to develop varieties with desired traits such as increased grain size or shorter reproductive cycles. Following studies would likely focus on further elucidating the genetic pathways and interactions responsible for the observed phenotypic changes. Through the use of advanced molecular techniques, including whole-genome sequencing and CRISPR-Cas9 gene editing, we can identify specific genes and alleles that contribute to these traits, offering insights into seed dormancy and potential methods for alleviating PHS.

The gene TraesCS1D03G0417700 (alpha-galactosidase) exhibited significant discrepancies between transcriptomic and qPCR results, potentially stemming from inherent limitations within both methodologies. qPCR measurements hinge on the abundance of specific gene fragments, while transcripts originating from different regions of the same gene can display substantial variation due to post-transcriptional regulation or alternative splicing [42]. Similarly, transcriptome sequencing accuracy may falter due to allelic effects or alternative splicing events. This vividly underscores the necessity and rationality for focusing on holistic changes in omics research, which could effectively mitigate the potential biases inherent in examining individual components.

## Conclusions

The wheat mutant 1813WH exhibited significantly enhanced seed dormancy (germination index 4.57 ± 3.48% vs. 42.77 ± 10.79% in wildtype) and longevity, representing a novel genetic resource for dissecting the regulatory network of these traits. Integrated multi-omics analysis revealed that elevated glutathione (9.8-fold higher) and raffinose (3.5-fold higher) contents, coupled with suppressed TCA cycle activity, collectively contribute to dormancy and longevity—providing evidence in wheat for the coordinated role of antioxidant metabolism, RFO accumulation, and energy metabolism in these processes. Practically, 1813WH offers valuable material for breeding PHS-resistant wheat, as its enhanced dormancy directly mitigates yield/quality losses from pre-harvest sprouting. While associated traits (reduced tillering, prolonged reproductive period) require optimization, this mutant highlights the potential to balance beneficial dormancy/longevity with agronomic performance, guiding future crop improvement strategies.

## Materials and methods

### Ethyl methanesulfonate (EMS) mutagenesis

The wheat cultivar “Long 13–3778” was obtained from the Heilongjiang Academy of Agricultural Sciences. Select healthy seeds of wild-type wheat “Long 13–3778”, sterilize with 70% ethanol and 2% sodium hypochlorite, then soak in 0.4% EMS solution at 25℃ in the dark for six hours. After treatment, rinse thoroughly with running water for eight hours, germinate and sow to harvest M_1_ seeds. The M_2_ mutant pool comprised of 5,600 mutant lines were acquired. Screen M_2_ generation for low germination index (GI) lines, self-purify to M_3_ generation to obtain genetically stable 1813WH mutant.

### Field management

The wild type and 1813WH mutant line were planted in an experimental plot at Inner Mongolia Agricultural University, Hohhot, China in April. The field management followed the local cultivation practices, with row length and spacing being 150 cm and 20 cm, respectively. Each line was sown in 10 rows.

### Phenotype assay

The phenotypes for the wild type and 1813WH mutant line were categorized and measured based on biological attributes, including morphological traits, yield-related traits, and growth period traits, with detailed protocols as follows. Statistical analysis was performed using ANOVA to evaluate differences between the wild type and 1813WH for all measured traits.

Morphological traits: This category includes plant height, internode length, stem thickness (including the length and thickness of the stem below the spike), grain length, and grain width. Measurements were conducted at the physiological maturity stage to minimize growth-stage variables. Tools used included a tape measure (1 mm precision), ruler (1 mm precision), vernier caliper (0.1 mm precision), and electronic caliper (for grain dimensions). In detail, plant height was measured from the base of the plant to the top of the main stem spike (excluding awns) using a tape measure. For internode length, stems were dissected into the first, second, and third internodes (from the base upward), and each internode length (distance between two adjacent nodes) was measured with a ruler. Stem thickness (including internodes and the stem below the spike) was measured at the midpoint of each internode using a vernier caliper (averaging transverse and longitudinal measurements). The above traits were measured from 10 randomly selected plants in each plot, and the mean values were used for subsequent analysis. Grain length and width were measured by aligning 10 randomly selected plump grains (from the 10 main-stem spikes) in a straight line. Total length/width of the aligned grains was measured with a ruler, and individual grain dimensions were calculated by dividing the total by 10 (*n* = 10 replicates).

Yield-related traits: This category comprises effective tillers, number of spikelets, grains per spike, and 1000-grain weight, measured at the maturity stage (when grains hardened and color stabilized). Effective tillers were counted as the number of tillers forming effective spikes (spike length ≥ 5 cm with ≥ 10 plump grains) per plant, with 10 replications (*n* = 10). The number of spikelets was determined by counting all spikelets (including degenerated ones, marked separately) from the base to the top of 10 randomly selected main-stem spikes. Grains per spike were calculated by stripping spikelets from these 10 spikes and counting plump grains (excluding shrunken ones) per spike. The 1000-grain weight was measured by randomly selecting 1000 plump grains from the harvested samples, weighing them with an electronic balance for ten replicates.

Growth period traits: This category is for the reproductive period, measured from the booting stage (when the young panicle was fully developed and the flag leaf unfurled) to the mature stage (when 50% of spikes turned yellow and grain moisture content ≤ 15%).

Germination index: fully mature seeds were collected, and the GI value of the mutant and the wild type was evaluated following the formula below:

$$\:\frac{100\times\:\sum\:{[G}_{t}\times\:{(D}_{s}+1-{D}_{t}\left)\right]}{{G}_{s}\times\:{D}_{s}}$$

Gt: number of germinated seeds of the day.

Gs: total number of germinated seeds.

Dt: day of germination.

Ds: total days of germination.

### Controlled deterioration tests

Newly harvested seeds were used as experimental materials. For each treatment group, 50 seeds were selected. The seeds were subjected to aging treatment under controlled conditions of 90% relative humidity and 40℃ temperature, with aging durations set at zero, two, five, and eight days, respectively. After undergoing the specified aging periods, germination tests were conducted for each group corresponding to different aging days. During the germination period, the number of germinated seeds was recorded daily from day one to day seven. Each treatment was replicated three times to ensure experimental reliability.

### Determination of glutathione, glutathione disulfide, and raffinose content

Gglutathione (GSH), glutathione disulfide (GSSG), and plant raffinose contents were determined using commercial kits with spectrophotometric detection, following the manufacturers’ protocols: G0206W Glutathione Kit (Suzhou Grace Biotechnology Co., Ltd., China), G0207W Glutathione Disulfide Kit (Suzhou Grace Biotechnology Co., Ltd., China), and MM-6,354,201 Plant Raffinose ELISA Kit (Jiangsu Enzyme Immunoassay Industry Co., Ltd., China). Fully matured seeds were used for all assays.

### RNA-sequencing and transcriptome-metabolome analysis

The collected grains at dough stage were quickly frozen in liquid nitrogen and stored at −80℃ for transcriptome and metabolomics analysis. Total RNA extraction, library construction, and sequencing were performed by Metware Biotechnology Inc. (Hubei, China). HISAT2 was used for mapping the clean reads to the Chinese Spring reference genome IWGSC CS RefSeq v2.1 (https://www.ncbi.nlm.nih.gov/datasets/genome/GCF_018294505.1). Differentially expressed genes (DEGs) were screened using DESeq2 [43, 44] and the false discovery rate (FDR) value were calculated. The screening conditions were set as log2FC value ≥ 1 and FDR < 0.05. Gene enrichment was performed based on GO (https://geneontology.org) and KEGG (https://www.kegg.jp) analysis.

As for the widely targeted metabolomics experiments, sample freeze-drying and processing as well as UPLC-MS/MS detection and data processing were conducted by Metware Biotechnology Inc. (Hubei, China). Differentially expressed metabolites were screened based on the variable importance in projection (VIP) value obtained from the OPLS-DA model, followed by hypothesis testing and fold change (FC) evaluation. Metabolites with VIP value > 1, P value <0.05 and FC ≥ 2 or ≤ 0.5 were designated as differentially expressed.

### qPCR analysis

For qPCR validation, the same RNA samples used in the RNA sequencing analysis were employed. Eight representative genes were selected, and specific qPCR primers were designed for each gene. The expression levels of these genes were determined using the 2^−ΔΔCT^ method [45].

The heatmaps represent the differentially expressed genes. W1-W3 and M1-M3 represent the gene expression in the wildtype and the mutant, respectively, of the enzymes in the corresponding metabolism pathways. The block icons marking the metabolites represent the Log2 FC value of the corresponding metabolite of 1813WH compared to the wild type. The gray block represents no significant differences. The legends represent gene expression (up) and the Log2 FC value of the metabolites (down). The abbreviations represent the enzymes of the corresponding pathways. AP: aminopeptidase; GS: glutathione synthase; GP: glutathione peroxidase; GR: glutathione reductase; GST: glutathione S-transferase. The detailed differentially expressed genes and metabolomic data are listed in the Supplementary Tables 3, 4.

The captions are identical to those in Fig. 8. The substances indicated in red text represent those that were not detected. RS: raffinose synthase; BF: beta-fructofuranosidase; AG: alpha-galactosidase; IAG: inositol 3-alpha-galactosyltransferase; UGE: UDP-glucose 4-epimerase; UGU: UTP-glucose-1-phosphate uridylyltransferase. The detailed differentially expressed genes and metabolomic data are listed in the Supplementary Tables 3, 4.

The captions are identical to those in Fig. 8. The substances indicated in red text represent those that were not detected. MDH1: malate dehydrogenase; fumAB: fumarate hydratase, class I; SDHA: succinate dehydrogenase (ubiquinone) flavoprotein subunit; sucB: 2-oxoglutarate dehydrogenase E2 component (dihydrolipoamide succinyltransferase); sucA: 2-oxoglutarate dehydrogenase E1 component; IDH1: isocitrate dehydrogenase; ACO: aconitate hydratase; ACLY: ATP citrate (pro-S)-lyase; gltA: citrate synthase; pckA: phosphoenolpyruvate carboxykinase (ATP). The detailed differentially expressed genes and metabolomic data are listed in the Supplementary Tables 3, 4.

## Supplementary Information

Below is the link to the electronic supplementary material.

Supplementary Material 1.

Supplementary Material 2.

## Data Availability

The RNA-Seq data is available in the NCBI Sequence Read Archive repository, https://www.ncbi.nlm.nih.gov/sra/PRJNA1290821. Other data supporting the conclusions of this article is included within the article and its additional files.
