# Supplementary figures and images for: Characterization of a wheat mutant line 1813WH presenting increased seed dormancy and longevity, and reduced pre-harvest sprouting
Source: BMC Plant Biol. 2025 Nov 13;25:1563. doi: 10.1186/s12870-025-07651-y (PMC12613733; doi:10.1186/s12870-025-07651-y)

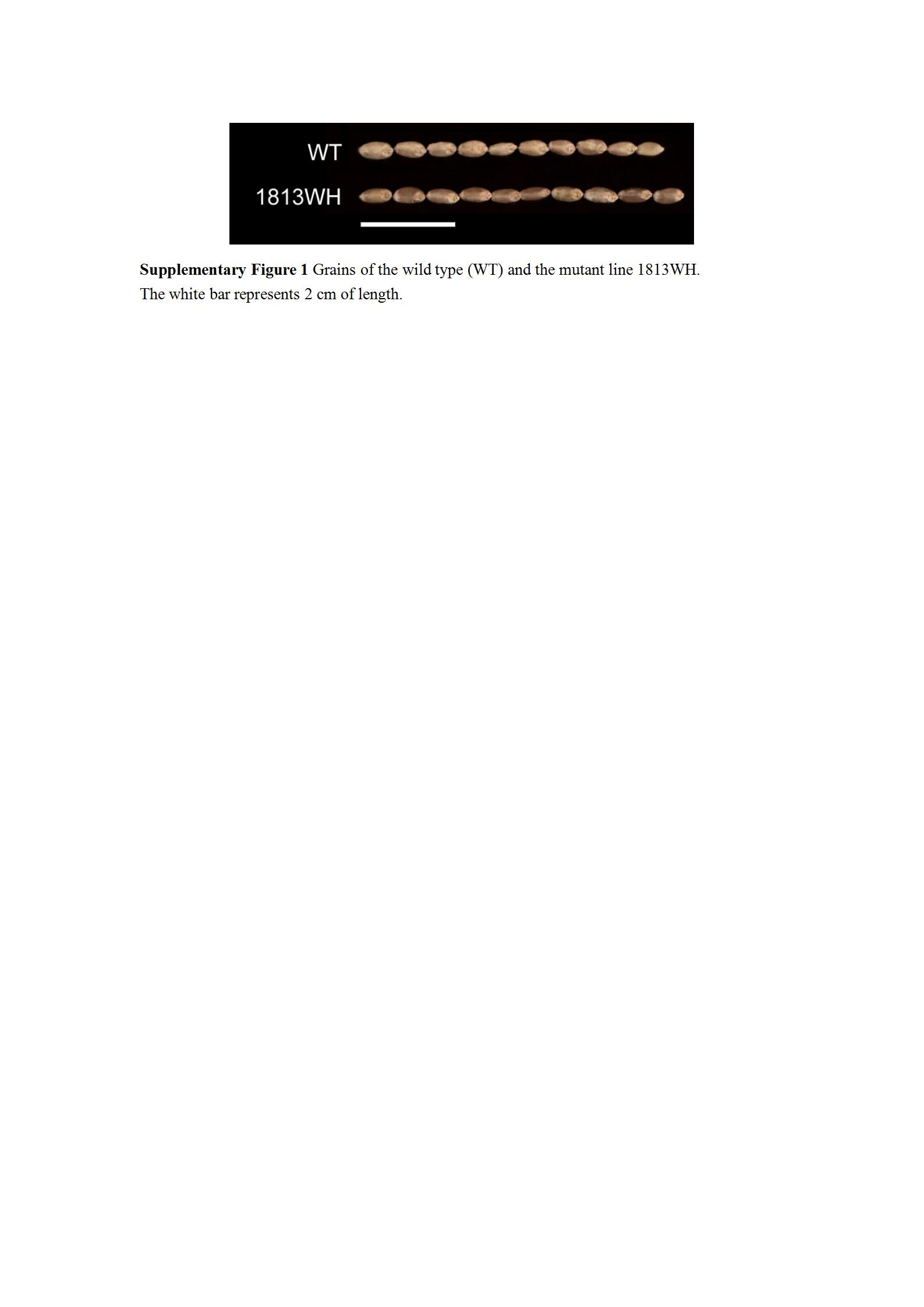

Supplement: Supplementary file 2 — Supplementary Material 2. [file 12870_2025_7651_MOESM2_ESM.jpg]
